# Rockwall and S. E. Collin Co. Med. Society

**Published:** 1886-07

**Authors:** 


ANOTHER STILL.—We are indebted to Dr. R. A. Taylor, of Millwood, Collin county, for a copy of the Constitution and By-laws of "The Rockwall and S. E. Collin County Medical Association," an organization recently effected, and of which Dr. R. A. Taylor is President, and Dr. H. W. Manson is Secretary.

